# Biomechanical analysis of the lumbar spine on facet joint force and intradiscal pressure - a finite element study

**DOI:** 10.1186/1471-2474-11-151

**Published:** 2010-07-05

**Authors:** Ching-Sung Kuo, Hsuan-Teh Hu, Ruey-Mo Lin, Kuo-Yuan Huang, Po-Chun Lin, Zheng-Cheng Zhong, Mu-Lin Hseih

**Affiliations:** 1 Department of Civil Engineering, National Cheng Kung University, Tainan, Taiwan; 2 Center for General Education, Nan Jeon Institute of Technology, Yenshui, Taiwan; 3 Department of Orthopaedics, National Cheng Kung University Hospital, Tainan, Taiwan; 4 National Cheng Kung University Hospital Dou-Liou Branch, Douliou, Taiwan; 5 Institute of Clinical Medicine, College of Medicine, National Cheng Kung University, Tainan, Taiwan; 6 Department of Physical Therapy and Assistive Technology, National Yang-Ming University, Taipei, Taiwan

## Abstract

**Background:**

Finite element analysis results will show significant differences if the model used is performed under various material properties, geometries, loading modes or other conditions. This study adopted an FE model, taking into account the possible asymmetry inherently existing in the spine with respect to the sagittal plane, with a more geometrically realistic outline to analyze and compare the biomechanical behaviour of the lumbar spine with regard to the facet force and intradiscal pressure, which are associated with low back pain symptoms and other spinal disorders. Dealing carefully with the contact surfaces of the facet joints at various levels of the lumbar spine can potentially help us further ascertain physiological behaviour concerning the frictional effects of facet joints under separate loadings or the responses to the compressive loads in the discs.

**Methods:**

A lumbar spine model was constructed from processes including smoothing the bony outline of each scan image, stacking the boundary lines into a smooth surface model, and subsequent further processing in order to conform with the purpose of effective finite element analysis performance. For simplicity, most spinal components were modelled as isotropic and linear materials with the exception of spinal ligaments (bilinear). The contact behaviour of the facet joints and changes of the intradiscal pressure with different postures were analyzed.

**Results:**

The results revealed that asymmetric responses of the facet joint forces exist in various postures and that such effect is amplified with larger loadings. In axial rotation, the facet joint forces were relatively larger in the contralateral facet joints than in the ipsilateral ones at the same level. Although the effect of the preloads on facet joint forces was not apparent, intradiscal pressure did increase with preload, and its magnitude increased more markedly in flexion than in extension and axial rotation.

**Conclusions:**

Disc pressures showed a significant increase with preload and changed more noticeably in flexion than in extension or in axial rotation. Compared with the applied preloads, the postures played a more important role, especially in axial rotation; the facet joint forces were increased in the contralateral facet joints as compared to the ipsilateral ones at the same level of the lumbar spine.

## Background

The lumbar spine is a part of the human body that is frequently activated during daily life. This consequently leads to a high incidence of disc problems, such as herniated disc, sciatica, and low back pain. Such disorders may arise from the wide range of motion in the lumbar spine, improper posture during the lifting of heavy objects, or maintaining an irregular posture for a long period of time. Up to now, many finite element (FE) simulations [1-9] as well as in vivo or in vitro studies [10-15] have been conducted for biomechanical analyses of the lumbar spine. However, most of the previous FE studies have used simplified models such as a quarter of the vertebrae and discs [1], a half of the vertebrae [2,4], or a lumbar spine model with a regular shape [3,5-8]. Additionally, many of the models have some asymmetry in the geometry with respect to the sagittal plane, it will undoubtedly reflect the asymmetric responses more or less to the left and right joints unless the model has been set up for symmetric simulation purposes. Because there is a lack of information regarding spine geometry, little processing to take into account irregular spine shapes, and no standard procedure to create a high quality biomechanical FE model, the simplifications made in the aforementioned models would adversely affect the results of the FE simulation.

In a real human spine, the geometry is different at each spinal level, such as the curvature of the facet joint, the dimensions of the vertebrae, and the height of the vertebral discs. Even for individuals of a similar stature, variability exists in vertebral responses in the human spine. Therefore, investigators are interested to know how the detailed geometry of the lumbar spine, which is composed of highly irregular posterior parts, affects its biomechanical behaviour. In addition, the discs at levels L4/L5 and L5/S1 are the sites that appear to be most associated with clinical problems and the development of spinal diseases. Furthermore, elucidating how the posture or loading mode influences the biomechanical behaviour at such levels is also of interest to researchers.

This study mainly takes into account the facet force and intradiscal pressure, which have a significant influence on human spine health, and have been considered by many researchers. For example, Shirazi-Adl et al. [1] used a three-dimensional nonlinear finite element study based on in vitro measurements to study the intradiscal pressure when under compressive loads. Lee et al. [2] indicated that nucleus pressure depends on the magnitude of compressive force rather than the loading rate (i.e. the impact force). In addition, Wang et al. [3] partly explored the effect of loading rates on intradiscal pressure using a viscoelastic finite element model of the L2/L3 motion segment, and found that the peak intradiscal pressure increased by 5.3% and 12.4% at the medium and fast loading rates, respectively, over the slow rate. They also indicated that the effect of posture on facet joint forces is more significant than that of loading rate. Rohlmann et al. [16] created a three-dimensional finite element model of the lumbar spine and reported that bone fusion affects intradiscal pressure in the adjacent intervertebral discs for extension. Zander et al. [17] found that an additional dynamic fixator below a rigid implant does not exert much influence on intradiscal pressure, but that it does reduce facet joint forces for axial rotation at its insertion level, and the hypothesis that intradiscal pressure is reduced by a dynamic implant could not be corroborated by their results. Rohlmann et al. [18] indicated that, compared to an intact spine, a dynamic implant reduces intradiscal pressure in a healthy disc for the purpose of extension and standing, and decreases facet joint forces at the implant level. They also found that some calculated parameters mostly represent trends, and due to the simplifications and assumptions necessary to create a finite element model of the lumbar spine, the absolute values are not always very precise. Moreover, Shirazi-Adl and Parnianpour [19] noted that the facet joint forces exhibited asymmetric behaviour in the left and right facet joints. In the Shirazi-Adl's study [20], a wrapping-element model to deal with large compression loads was built, in which it was emphasized that the ligamentous lumbar spine devoid of musculature could barely resist large compressive forces. The present study developed an FE model of the lumbar spine with a realistic geometric shape, particularly in the posterior bony parts of the spine, to simulate the lumbar spine subjected to several loading conditions and approached the above mentioned claim, in order to investigate to what extent the real geometry of the lumbar spine is affected by asymmetry. We also compared the effects of symmetric postures, such as left and right axial rotations, on the facet joint forces at various levels of the lumbar spine to explore the extent to which they were affected. In addition, we investigated the effect of various postures on intradiscal pressures in the nuclei pulposi.

## Methods

### CT scanning, image processing, and bony outline smoothing

**Figure 1:**
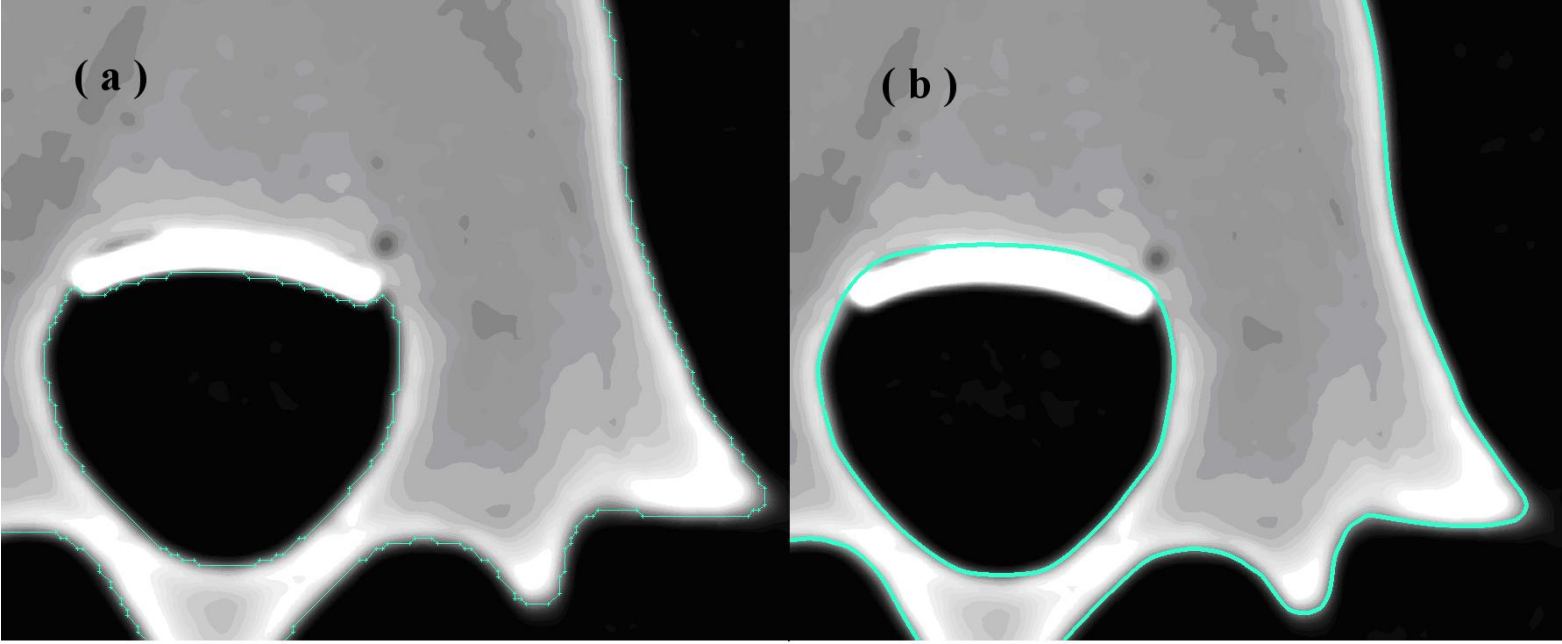
The (a) jagged and (b) smooth contour lines of a DICOM image.

Computed tomography (CT) images with a slice distance of 1 mm (512 × 512 resolution, 16-bit, and a pixel size of 0.3516 mm × 0.3516 mm) were acquired from scanning a specimen of a lumbar spine model. Bony boundary outlines were depicted from each DICOM image filtered using a gray value threshold. These contour lines could not be stacked efficiently into a better surface model owing to their sawtooth shapes (Figure 1(a)). Through further processing of the bony outlines (Figure 1(b)) and the use of 3D-DOCTOR software, a smooth surface model was created.

### Preprocessing of the FE model

The stereolithography (STL) format surface model thus obtained was, however, still not suitable for FE analysis; it required further preprocessing in order to detect whether high aspect ratio elements and gaps were left in the model, and also to adjust the element side between 1 mm and 3 mm, so as to retain the accurate geometry of the spine. The relative changes in vertebral volume before and after the smoothing process using PATRAN are listed in Table 1 for comparison. There was on average only a 1.95% volume change after the smoothing process. As a consequence of this improvement not only was the realistic geometry of lumbar spine retained but the performance efficiency of this model on computers was also maintained. In this study, the contact behaviour of facet joints was simulated, with the coefficient of friction set to 0.1-similar to that used by Polikeit et al. [21] - and the effect of the capsular ligaments was incorporated with that of the facet joints modelled by CONTACT PAIR SURFACE elements.

**Table 1:** Relative changes in vertebral volume, before and after smoothing with PATRAN

Vertebra	3D-Doctor	PATRAN	Relative
	**(mm**^**3**^)	**(mm**^**3**^)	Error (%)
L1	55967.03	54740.16	2.19
L2	62952.83	61648.31	2.07
L3	62931.89	61842.08	1.73
L4	70881.32	69461.11	2.01
L5	65987.41	64817.10	1.77
Total	318720.48	312508.76	1.95

### Materials and element types

A vertebra consists of a cancellous bone, cortical shell (thickness, 0.35 mm), posterior bone, and endplates (thickness, 0.5 mm). A disc is composed of a nucleus pulposus, annulus fibrosus, and annulus ground substance. This study adopted linear and isotropic material properties for most spinal components such as the cancellous bone, cortical shell, posterior bone, endplate, annulus fiber layer, annulus ground substance, and nucleus pulposus (Table 2), whereas spinal ligaments were modelled as bilinear materials (Table 3).

**Table 2:** Properties of the materials used in this study

Material	Young's modulus	Poisson ratio	Reference
	E (MPa)	ν	
**Vertebra**			
Cortical bone	12000	0.3	[21,27-32]
Cancellous bone	100	0.2	[28-32]
Endplate	12000	0.3	[12,27,29]
Posterior elements	3500	0.25	[18,21,30,33,34]
**Disc**			
Nucleus pulposus	1	0.4999	[16,30,35,36]
Annulus ground substance	4.2	0.45	[27-30,36-38]
Fiber			
(inner)	360	0.3	[29,39] 360~550(E)
(outer)	550	0.3	[27,40] 450(E)

**Table 3:** Properties of the ligaments used in this study [29,41,42]

Ligament	ALL	PLL	LF	ISL	SSL	TL
Elastic modulus (small strain) **(MPa)**	7.8	10	15	10	8	10
Transition strain **(%)**	12	11	6.2	14	20	18
Elastic modulus (large strain) **(MPa)**	20	50	19.5	11.6	15	59
Cross-sectional area **(mm**^**2**^**)**	53	16	67	26	23	1.8
Length **(mm)**	13	11	19	13	11	22
Max. failure load **(N)**	510	384	340	130	200	70

**ALL**, Anterior Longitudinal Ligament

**TL**, Transverse Ligament

**SSL**, SupraSpinous Ligament

**PLL**, Posterior Longitudinal Ligament

**LF**, Ligamentum Flavum

**ISL**, InterSpinous Ligament

In order to preserve the original geometry of the lumbar spine, the current model used solid tetrahedral linear elements (C3D4, ABAQUS) instead of hexahedral ones to simulate the irregular posterior bone, cancellous bone, and annulus ground substance. For the nucleus pulposus, near incompressible tetrahedral elements were employed. Cortical shell (bone), endplate, and annulus fiber layers were modelled by triangular shell elements (M3D3) with element side in the range of 1 mm to 3 mm, and ligaments were modelled as narrow strip-shaped membrane elements (M3D3) under the control of no resistance in compression by the user-subroutine in ABAQUS (Ver. 6.5-1). The element types and number of elements used in the components of the spine are listed in Table 4.

**Table 4:** Element types and number of elements used in components of the spine

Component	Element type	No. of elements					
		
		L1	L2	L3	L4	L5	
Cortical bone	M3D	385	3820	884	787	711	
Cancellous bone	C3D4	4511	4180	4543	4285	4140	
Endplate	M3D3	582	548	530	562	580	
Posterior bone	C3D4	11773	15064	13636	12102	10594	
		**L1/L2**	**L2/L3**	**L3/L4**	**L4/L5**	**L5/S1**	
		
Nucleus pulposus	C3D4	1048	954	992	988	833	
Annulus fibrosus	M3D3	639	788	742	727	818	
Annulus ground	C3D4	2079	2640	2447	2493	3201	
substance							

**Ligament**		**ALL**	**PLL**	**LF**	**ISL**	**SSL**	**TL**
**No. of elements**	M3D3	1544	592	552	335	250	710

### Loading and boundary conditions

The loading conditions consisted of an evenly distributed load of 300 N, 460 N, or 600 N as the upper body weight for the case of standing, as well as combinations of a preload of 300 N, 460 N, or 600 N; forward/backward bending moments of 5 Nm, 10 Nm, 15 Nm, and 20 Nm for flexion and extension; and left/right rotation moments of 5 Nm, 10 Nm, 15 Nm, and 20 Nm for axial rotation. All these moments were applied on the superior surface of the L1 vertebral body. The boundary conditions imposed were set with the nodes on the endplate of S1 constrained in all directions.

### Convergence test and validation

Although Ramos et al. [22] indicated that hexahedral quadratic elements appeared to be more stable and less influenced by the degree of refinement of the mesh when modelling a simplified proximal femur, their results from simulating a realistic proximal femur with first and second order tetrahedral and hexahedral elements did not demonstrate significant differences. We used the L1 vertebra for convergence test due to similar consideration and formulation for the other vertebrae, and measured the displacement of a reference point on the top surface of L1 vertebral body under a uniformly distributed load of 0.5 MPa. Five different amounts - 33797, 24190, 19012, 14939, and 12044 elements - were compared for their corresponding displacements. By setting the displacement of the L1 vertebra to 33797 elements as a reference value, the errors with the total number of elements were reduced - all were within 1.2%. In this model, we selected a total of 17719 elements for the L1 vertebra based on the small relative displacement error of 0.33%.

**Figure 2:**
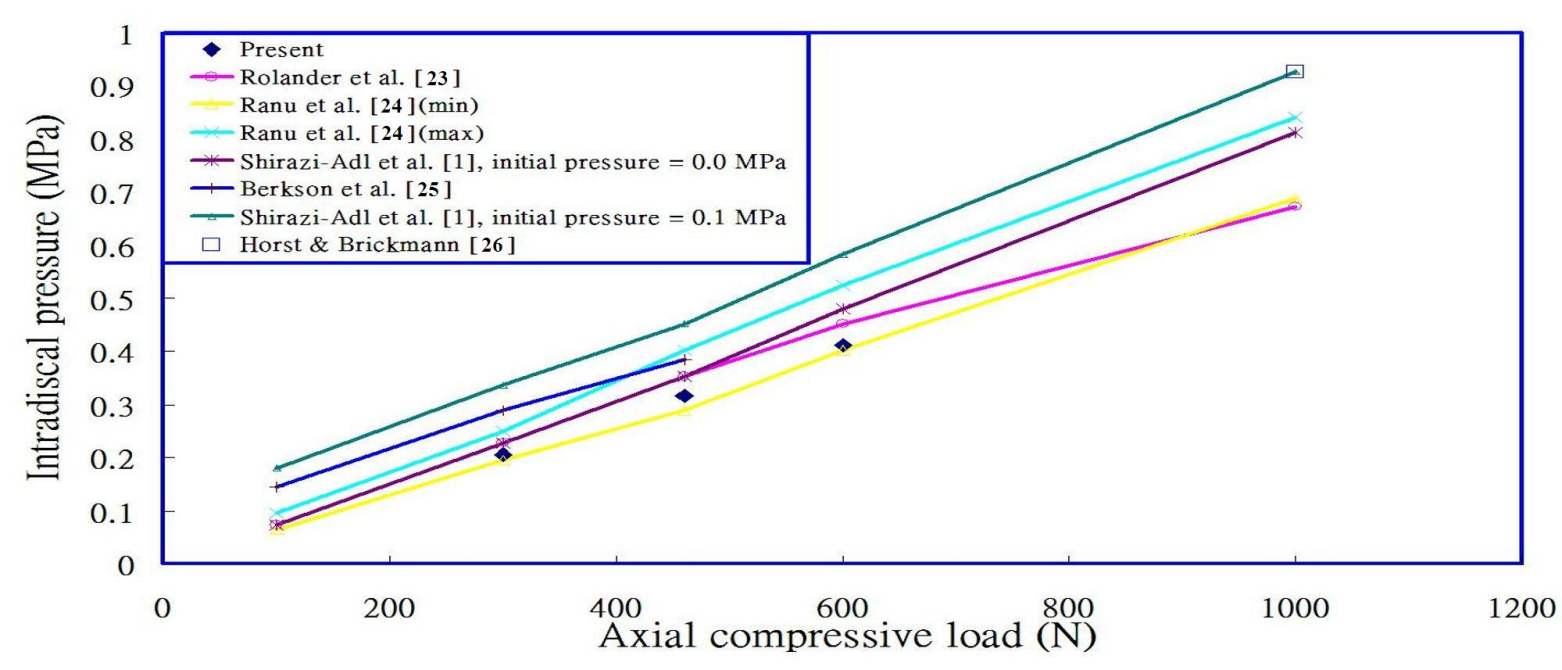
Comparison of the calculated results with previous studies.

**Figure 3:**
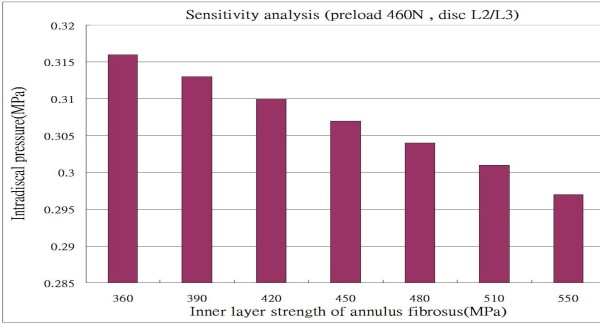
Sensitivity analysis for the intradiscal pressure versus the inner fiber strength, under preload 460N, at level L2/L3.

**Figure 4:**
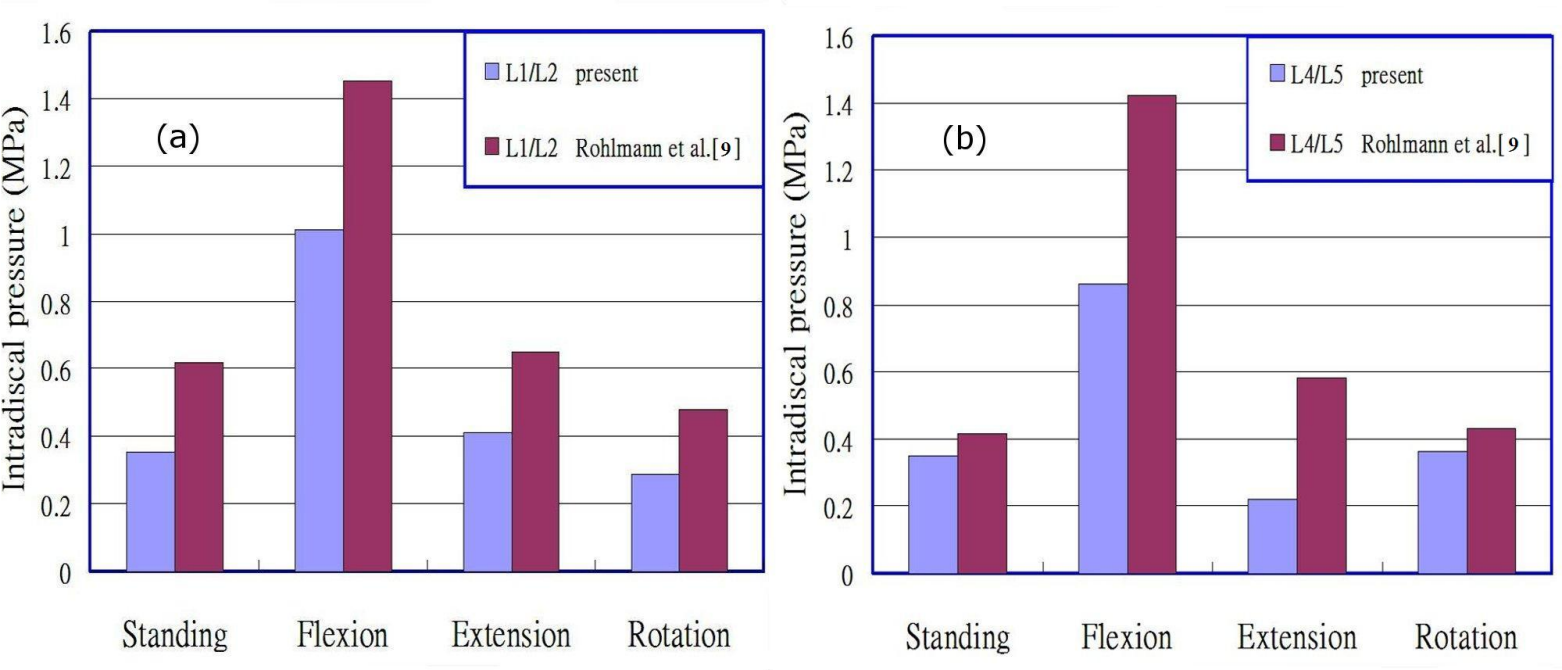
**The changes in intradiscal pressure in the (a) L1/L2 and (b) L4/L5 disc of present study and Rohlmann et al**. [9]**for the intact lumbar spine.**

To validate the constructed model, we compared the calculated intradiscal pressures of the L2/L3 disc, under the preload of 300N, 460 N and 600N respectively over the superior surface of the L1 vertebral body in a standing posture, with those reported in the literature [1,23-26] (Figure 2), and found that the linearity with compressive load is in agreement with their studies. Although the calculated data in this study appears to be relative lower than most of the previous results, it could be interpreted reasonably from the fact that the stronger homogeneous annulus fiber elements were used in this study, and the sensitivity analysis for the intradiscal pressure versus the inner fiber strength at the level L2/L3, under preload 460N, is shown in Figure 3. The changes in intradiscal pressures at various levels in different postures also exhibit the similarity when compared to the research by Rohlmann et al. [9] as shown in Figure 4.

## Results

The numerical results were principally concerned with facet joint forces at various levels of the lumbar spine and the intradiscal pressures in the discs under different preloads. In addition, the von Mises stresses/strains in the lumbar spine in the standing position were also studied for analysis.

### Von Mises stress/strain

**Figure 5:**
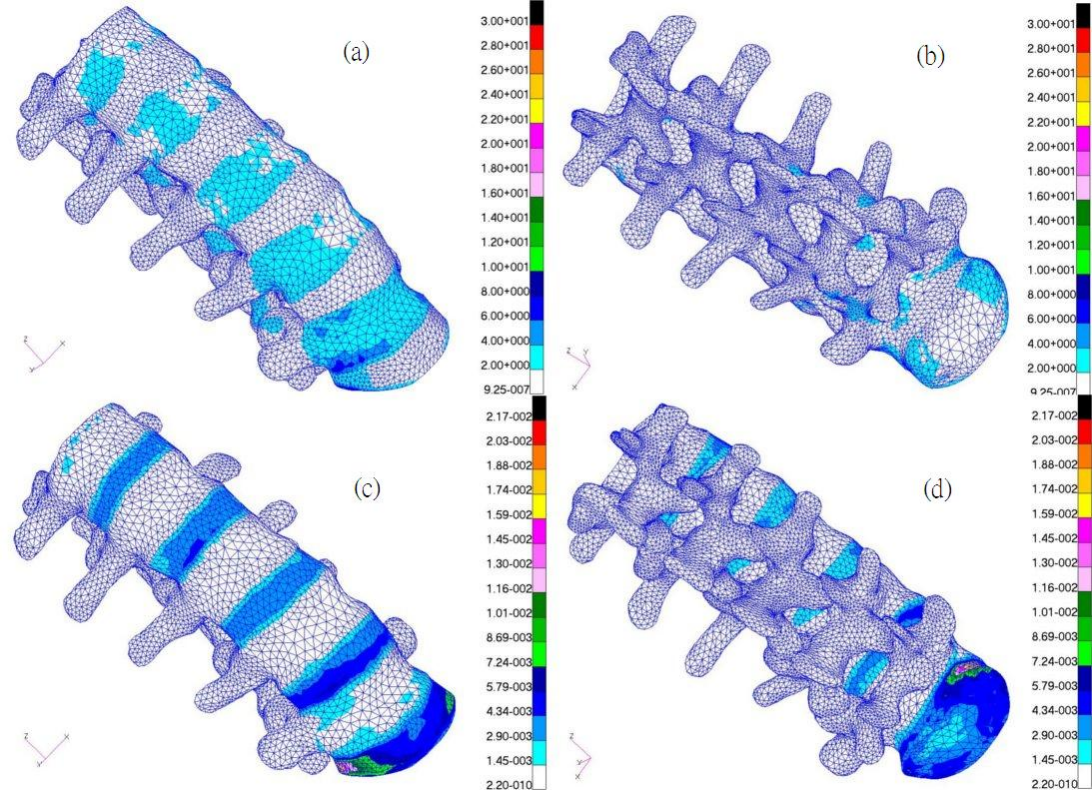
(a) Front view/(b) Back view of von Mises stress distribution and (c) front view/(d) back view of von Mises strain distribution in the lumbar spine (without ligaments) under an evenly distributed load of 460 N over the superior surface of the L1 vertebral body in a standing posture.

It was observed that the von Mises stress increased steadily downward along the lumbar spine (Figures 5(a) and 5(b)), and that the lumbar vertebral bodies were the major load-bearing parts of the spine sustaining physiological loadings. The recorded values varied from approximately 4.61 MPa to 9.82 MPa in the L5 vertebral body, and reached a maximum value of 9.82 MPa at the lower right rim of the lateral cortical shell of the L5 vertebral body. Undoubtedly, the discs are the major spinal components for the absorption of impact energy, particularly during the loading period. Through deformations of the discs, loads can be transmitted gently in order to prevent spinal injury. As in the case of stress, there was a trend of downwardly increasing strain (Figures 5(c) and 5(d)).

### Facet force

**Figure 6:**
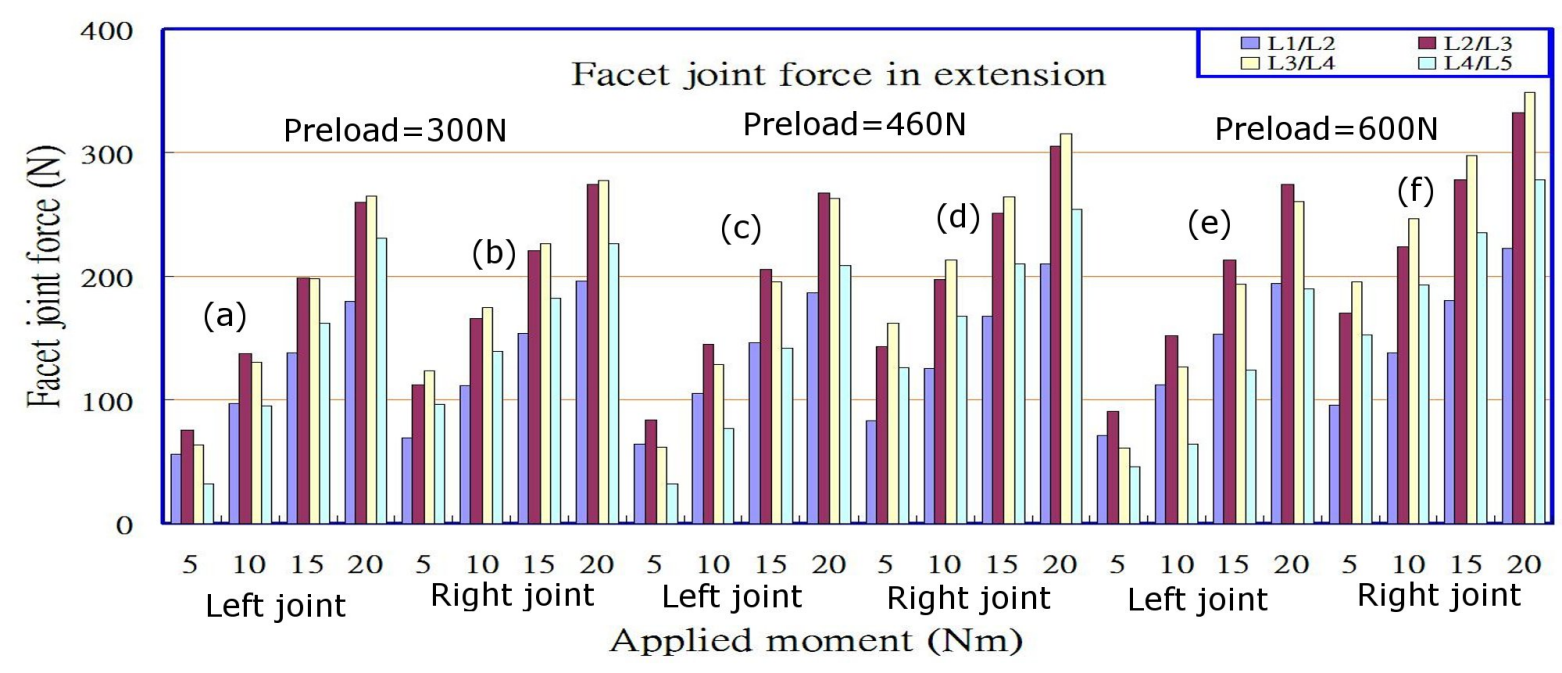
Facet joint forces at various levels under different combinations of preloads and loadings in extension.

**Figure 7:**
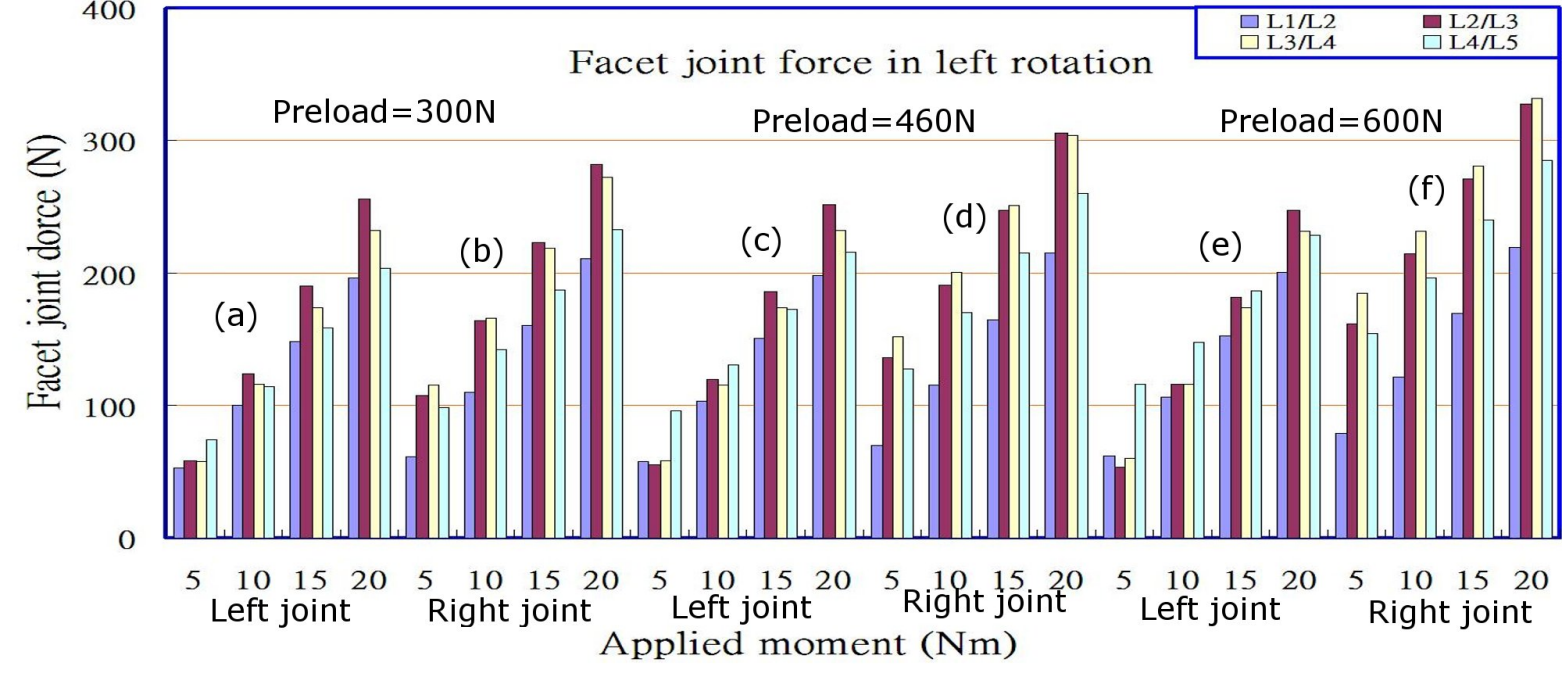
Facet joint forces at various levels under different combinations of preloads and loadings in left rotation.

**Figure 8:**
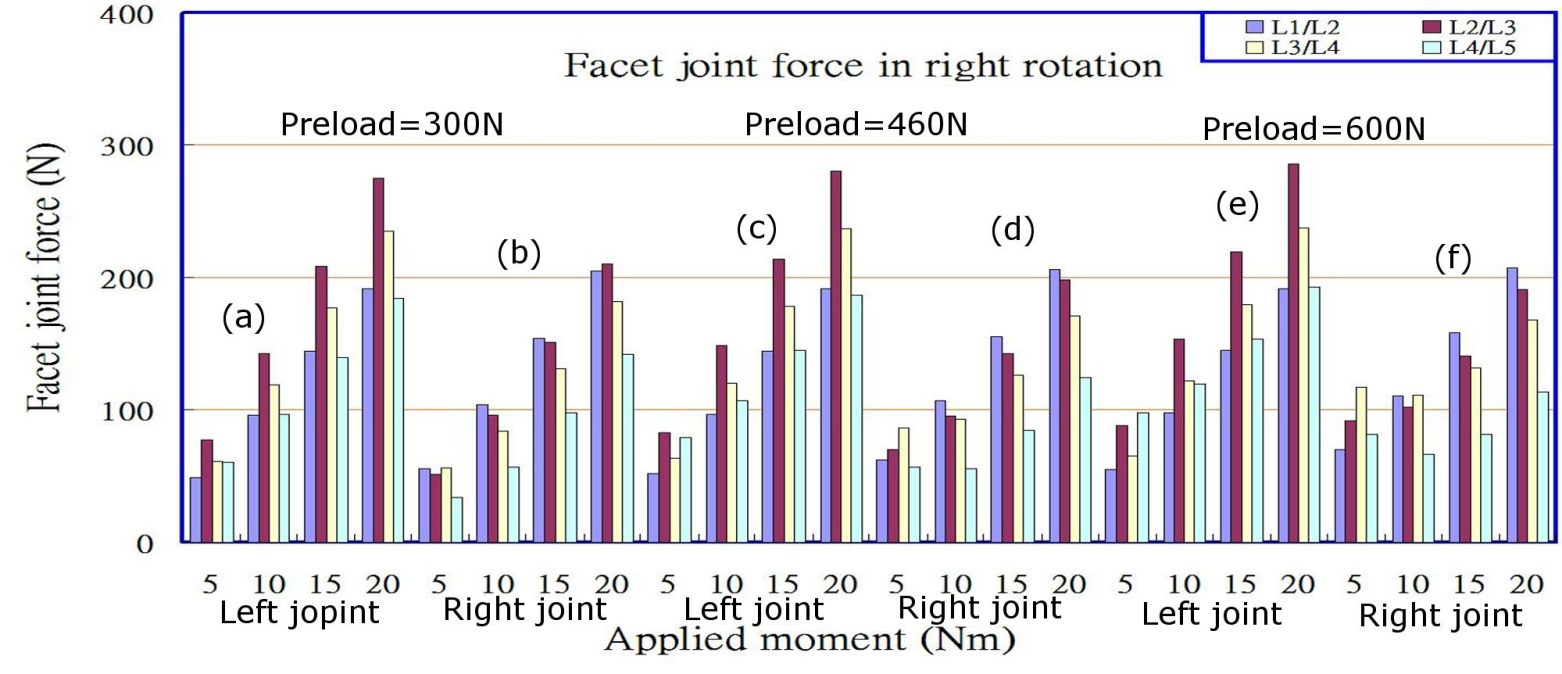
Facet joint forces at various levels under different combinations of preloads and loadings in right rotation.

The results shown in each of Figures 6, 7 and 8 were further divided into 3 groups based on the magnitude of the preloads (300 N, 460 N, and 600 N); these included 2 subgroups for the left and right facet joints at various levels under the loading of 5 Nm to 20 Nm in each group. For example, in the case of extension postures (Figure 6), under preloads of 300 N, 460 N, and 600 N and different loadings from 5 Nm to 20 Nm by an increment of 5 Nm, Figures 6(a) and 6(b), Figures 6(c) and 6(d), and Figures 6(e) and 6(f) respectively, denote the facet joint forces between the left and right facet joints.

The data shown in Figures 6, 7 and 8 indicate that the facet joint forces at various levels of the lumbar spine under different loadings, in extension, and with left/right axial rotations, were affected slightly by the preloads in the range of 300 N to 600 N, particularly in right rotation, and that they increased comparatively little (contrast Figure 6(a) with Figures 6(c) and 6(e); contrast Figure 6(b) with Figures 6(d) and 6(f); similarly for Figures 7 and 8). However, the differences of facet joint forces between the left and right facet joints varied with the preload in extension and left rotation, particularly in the right joints. It also appeared that there was an asymmetric behaviour between left and right rotations for both the left and right facet joints at each level, irrespective of the type of loading that was applied. This could be observed from that the magnitudes of facet joint forces (75.95 N and 63.42N in left joint at levels L2/L3 and L3/L4); however, at the corresponding levels, the values were 112.08 N and 123.31 N in the right joints under an extension moment of 5 Nm and a preload of 300 N. When the extension moments was increased from 5 Nm to 20 Nm, the results indicated that the facet joint forces had larger values at levels L2/L3 and L3/L4 than at levels L1/L2 and L4/L5 (Figures 6(a) and 6(b)), and increased with an increase in the applied moment. At the same level and under the same extension moment, facet joint forces in the right joints were larger than those in left joints (contrast Figures 6(b), (d) and 6(f) with Figures 6(a), (c) and 6(e), respectively; similarly for Figure 7). Furthermore, if the lumbar spine was loaded by a left rotation moment, the right (opposite) joint had a larger facet force than the ipsilateral (left) joint at the corresponding level (contrast Figure 7(a) with Figure 7(b); contrast Figure 7(c) with Figure 7(d), etc.), and vice versa for the case of right rotation (contrast Figure 8(a) with Figure 8(b); contrast Figure 8(c) with Figure 8(d), etc.). We therefore observed that applying bending or rotation moments to the lumbar spine played a more important role than the applied preload on the facet force.

### Intradiscal pressure

**Figure 9:**
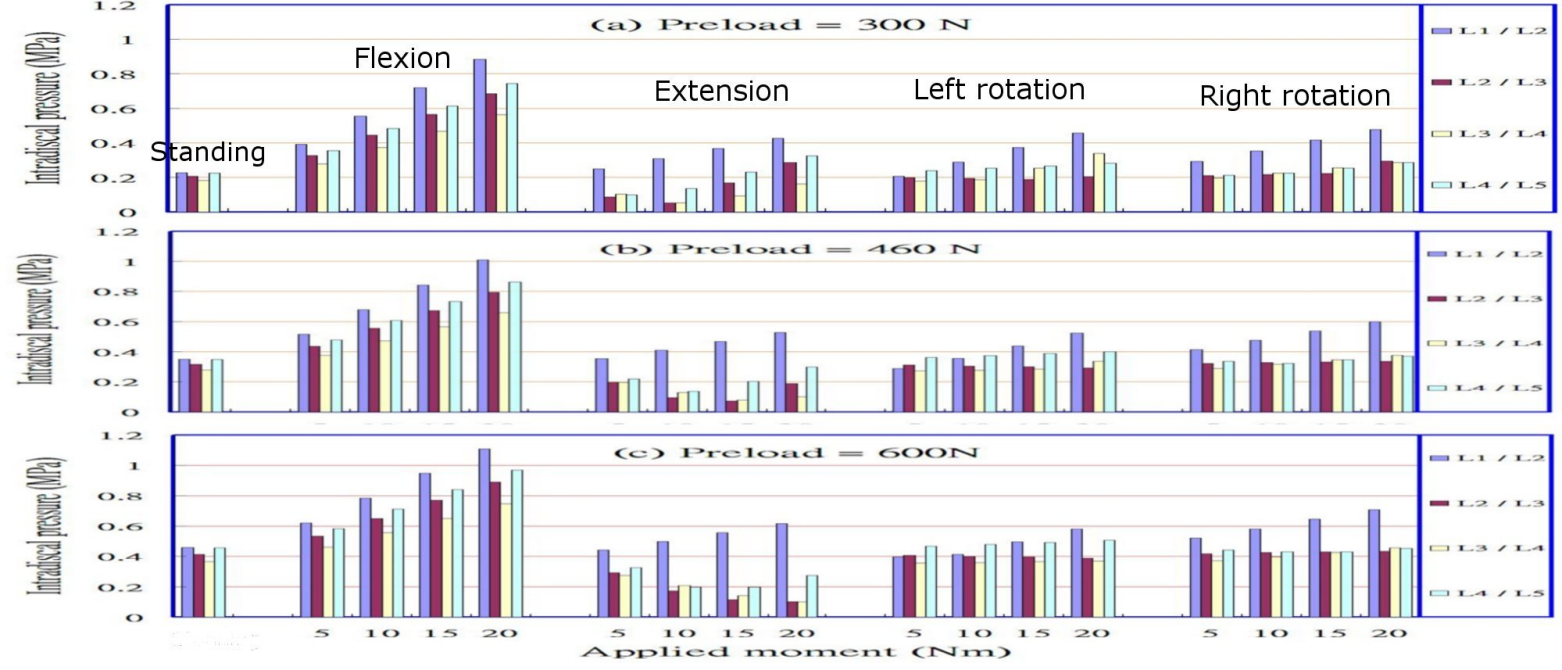
Intradiscal pressures at various levels of the lumbar spine under preloads of (a) 300 N, (b) 460 N, and (c) 600 N and different loadings, including forward/backward bending moments and left/right rotation moments of 5 Nm, 10 Nm, 15 Nm, and 20 Nm.

Figures 9(a), (b) and 9(c) show the intradiscal pressures of the nuclei pulposi at various levels under preloads of 300 N, 460 N, and 600N and at different loadings. From these figures, the calculated data for intradiscal pressures increased with preload. In an upright standing posture, the average pressure at all levels (L1/L2 to L4/L5) was approximately 0.2 MPa under a preload of 300 N, and was 0.324 MPa and 0.42275 MPa under preloads of 460 N and 600 N, respectively. In flexion, the pressure increased noticeably compared with other postures and had a value of approximately 0.9 MPa at level L1/L2 under a forward bending (flexion) moment of 20 Nm. In the case of left and right rotations, the intradiscal pressures were relatively larger at level L1/L2 than those at other levels under the same loading and different preloads. For the left rotation, levels L1/L2 and L4/L5 had values higher than levels L2/L3 and L3/L4. Level L2/L3 did not appear to be affected by the left/right rotation postures and maintained a value of approximately 0.2 MPa when subjected to a rotation moment of less than 15 Nm. Similarly results were obtained for level L3/L4 under a rotation moment of less than 10 Nm. As in the case of extension, initially the intradiscal pressures at levels L2/L3 and L3/L4 were reduced temporarily, then after the backward bending moment exceeded 10 Nm or 15 Nm, the pressures gradually increased.

## Discussion

In this study, we developed a realistic model preserving the complex geometry of the posterior parts of the lumbar spine in order to investigate the relevant biomechanical behaviour and to examine whether the asymmetric responses increase with loading, and if even larger loads amplify the effect of asymmetry. The facet geometry of our study model was obtained through stacking the bony outline of each DICOM image file from scanning a lumbar spine specimen (1 mm space apart in the CT series images), and there existed tiny asymmetries about the sagittal plane of the spine if we inspected the specimen carefully. Even when we examined the shapes of some of the investigated models, asymmetry also appeared to some extent. In addition, most of the related studies have hypotheses about symmetric behaviour. The spinal bones did not seem to remain absolutely symmetric during the growth period due to the frequent variations in the spinal development environment, and, as the vertebral bone of the spine is alive, it adapts itself to such changes. Because of the lack of the geometric dimensions used in the related experimental data, different loading and boundary conditions, various curvatures of the facet joints, and so on in the related research, it is difficult to make exact comparisons between our results and those of other studies. Therefore, in most instances, we present only the trends in the responses of the lumbar spine observed in the present analysis.

From Figures 5(a), (b), (c) and 5(d), it can be seen that the lower lumbar spine has a larger stress or strain distribution. From the data obtained in our simulation, the results show that the asymmetry is gradually more obvious with preloading in the right joints under extension posture if we compare Figures 6(b), (d) and 6(f), while there are few variations with preloading in the left joints seen in Figures 6(a), (c) and 6(e). Similarly for the case of left rotation, if we examine Figures 7(b), (d) and 7(f) and another group of Figures 7(a), (c) and 7(e), the same response modes appear. However, in Figures 8(a), (c) and 8(e) or Figures 8(b), (d) and 8(f), there are few changes in left or right facets, i.e., the asymmetry is not evident with preloading, but it increases with the applied moment. These results made us associate this asymmetry with the geometric defects in the right facet joint, no matter what the cause originated from the specimen geometry or the manual work in depicting the bony outlines. The asymmetry diminished if the lumbar spine rotated to the right and decreased the contact area between the right facet surfaces.

**Figure 10:**
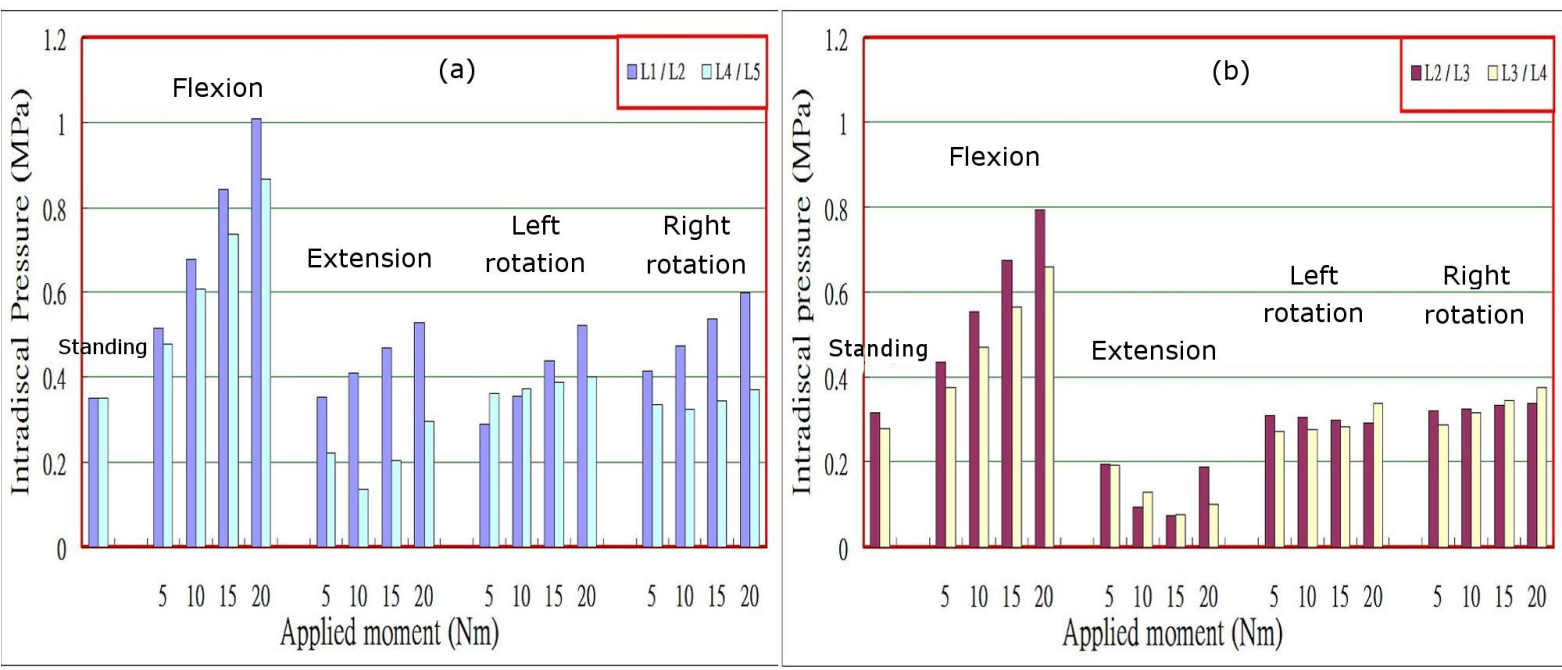
Intradiscal pressures at (a) levels L1/L2 and L4/L5, and (b) levels L2/L3 and L3/L4 under a preload of 460 N.

In order to validate the constructed model, we rearranged the results shown in Figure 9(b) by separating levels L1/L2 to L4/L5 for the intradiscal pressures of the lumbar spine under a preload of 460 N and different loadings into two parts: levels L1/L2 and L4/L5 in Figure 10(a), and levels L2/L3 and L3/L4 in Figure 10(b). The calculated data shown in Figures 10(a) and 10(b) appear to exhibit a trend similar to that for the intact lumbar spine reported in the literature [9,18], in which a total load of 460 N resulting from an applied load of 260 N representing the weight of upper body was adopted, together with a compressive follower load of 200 N representing the stabilizing effect of the local muscle forces. In addition to the above loadings, Rohlmann et al. [9] employed the following physiological loadings: (1) standing, 30° flexion (forward bending), 15° extension of the lumbar spine, and 6° torsion (axial rotation); and (2) standing, 30° flexion, 20° extension, and 10° torsion [18]. In the case of standing, the intradiscal pressures at levels L1/L2 and L4/L5 calculated in the present study were 0.351 MPa and 0.349 MPa, respectively. However, Rohlmann et al. obtained higher values of approximately 0.61 MPa and 0.58 MPa at the corresponding levels. This disparity can be attributed to an additional force in the erector spinae or the rectus abdominis, and/or the different manner of applying loads used in the previous studies, as well as the stronger homogeneous annulus fiber elements that were used in our study. An additional sensitivity analysis was conducted to validate our assumption, as shown in Figure 3. In the cases of other postures, intradiscal pressures had larger values in flexion rather than in extension and left/right rotations, particularly at level L1/L2. There was a similar trend of changes with loading in flexion, extension, and left/right rotations between the present analysis and that reported in the literature [9,18]. A further trend can be observed in Figure 10(b); namely, that the pressures at levels L2/L3 and L3/L4 were clearly reduced in the case of extension under moments of 5 Nm to 10 Nm or 15 Nm. This phenomenon might be interpreted as a variation in the curvature of the lumbar spine at different levels. The magnitude of the moments appeared to slightly alter the curvature of the spine at level L2/L3 or L3/L4 during the period of extension, whereas the pressure had a large value at level L1/L2. The latter observation could be related to the backward bending moment that was applied over the superior surface of the L1 vertebral body nearing the disc most closely at level L1/L2. In addition, the intradiscal pressures in the nuclei pulposi increased with increasing magnitude of preloads (Figures 9(a), (b) and 9(c)), and this appeared to be a reasonable outcome for the general physiological loading cases.

**Figure 11:**
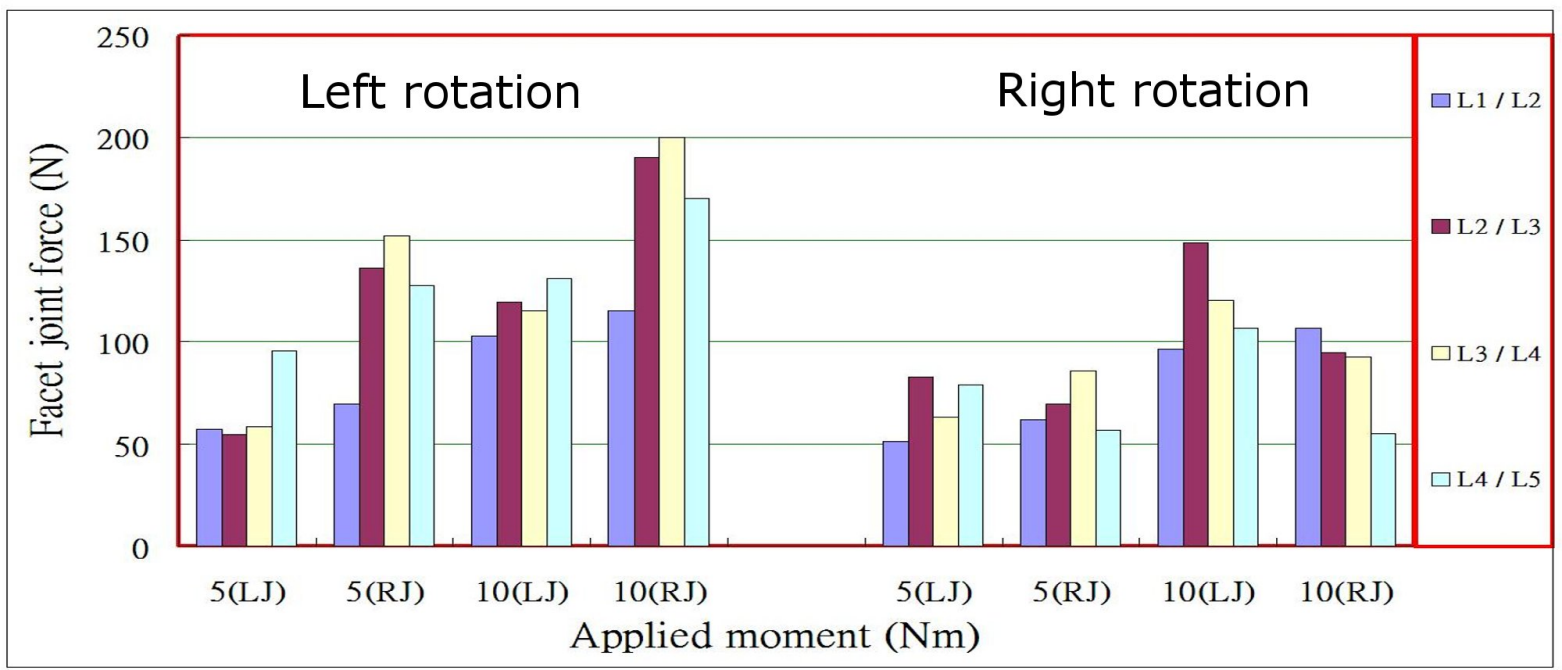
Facet joint forces at various levels under a preload of 460 N and left/right rotation moments of 5 Nm and 10 Nm, (L) and (R) denote left facet joint and right facet joint, respectively.

For the facet joint forces at various levels of the lumbar spine under a preload of 460 N and left/right rotation moments of 5 Nm and 10 Nm, Figure 11 indicates that forces increased with increasing axial rotation moment and were higher in the contralateral facet joints than in the ipsilateral joints, as reported by Shirazi-Adl's study [20], particularly for levels L2/L3 and L3/L4 in the case of left rotation. It was also observed that the facet joint forces in the left and right facet joints exhibited asymmetric behaviour in left/right rotation, as also reported by Shirazi-Adl and Parnianpour [19]. For example, the facet joint forces at levels L2/L3 to L4/L5 in the right joints under a left rotation moment of 5 Nm differed considerably from those at the corresponding levels in the same joints under right rotation. Similar patterns were observed for level L2/L3 in the left joint under a rotation moment of 10 Nm, and at levels L2/L3 to L4/L5 in the right joint under a rotation moment of 10 Nm.

In addition to the above arguments concerning the asymmetric response of facet joint forces, there were differences between left and right facet joints at each level of the lumbar spine for extension and left/right rotation. The facet force at level L3/L4 did not have a higher value than that at level L2/L3 until the backward (extension) moment reached 20 Nm (Figure 6(a)); however, in the right facet joints, the facet force at level L3/L4 always slightly exceeded that at level L2/L3.

As to the asymmetric behaviours in left and right rotation in our model, they could be due to the small inherent asymmetry in the specimen geometry or some manual errors in construction process of the bony outlines. The asymmetric behaviour due to the geometrical factor in the present model did not appear to be expressed as some mathematical relation, such as a linear relation, squared relation, or some combination of these. And the formation of the asymmetry was not attributed to a single factor, as geometrical factors, vertebral body dimensions, facet joints, endplates, pedicles, alignment of the vertebral bodies, or loading condition factors, like applied force, moment, torque, even the constraint (support) condition, for example, the lumbar spine with one side facet resection, would all affect the global asymmetric behaviour. How to quantify the asymmetric behaviour was thus really a complicated process that deserves further attention in future work. The effect of asymmetric loading of the facet joints with respect to the saggital plane in left and right rotation would be amplified with larger applied loads, because the magnitude of the moment (or torque) is the product of force and length of arm of force. In addition, the real physiological response or mechanical behaviour of the spine with activating components like soft tissues, ligaments, tendons, or muscles could differ to some extents from that of the FE model purely derived from the bony outline, without taking into account muscular tissues. Thus, how to improve the asymmetry might be another issue for future researchers to investigate, as the image processing of the bony outlines of the lumbar spine was a tedious manual task. Even if the specimen had inherent defects or the outlines of facet joints, which were depicted and modified by hand, were somewhat incorrect, the correction work cannot be finished without further FE preprocessing and execution by the FE program. From the anatomical point of view, our model was obtained through stacking the bony outlines of vertebral bones which were molded from a human cadaver which, to the best of the authors' knowledge, was without bony defects prior to the process of producing the lumbar specimen. In addition, unless there is sufficient spinal dimensions data available to make a statistical analysis and conclude that the real situation of most of the human spine is symmetric with respect to the sagittal plane of the spine, the tiny asymmetries about the sagittal plane of the spine can not be avoided in the model construction. When only the simulation data has been corrected and validated, it is hard to conclude whether the asymmetry is due to only the manual errors or the inherent defects. This issue deserves further attention in future research. As long as the model has few shifts from the symmetric geometry, and there is a larger applied load, without considering the effect of muscles balancing the left and right facet joints, the asymmetry effect does not seem to be easily removed.

To the best of the authors' knowledge, whether the asymmetry would alter the coupled motion or not is related to factors such as the speed of movement of the spine, the strength of muscles attaching to the spinal bones in individuals, forces in the erector spinae or rectus abdominis, the relative sliding smoothness of spinal joints and so on. So to some extent the asymmetry in the present model would make a difference to the action behaviour with regard to the sagittal plane and to the responses in left and right joints, and thus would also affect the coupled motion, which associates lateral bending in left and right directions with horizontal (axial) rotation.

## Conclusions

The results suggest that von Mises stresses/strains responded to a preload of 460 N with higher values in the lower part of the lumbar spine. Intradiscal pressures in the nuclei pulposi increased with preload and increased more noticeably with flexion than with extension or axial rotation. In extension postures, pressures were reduced at levels L2/L3, L3/L4, and L4/L5 under different preloads. With regard to the facet joint forces, forward/backward bending and left/right axial rotations produced asymmetric responses in the facet joints. Left axial rotation resulted in a larger facet force in the contralateral (right) facet joint than that in the ipsilateral (left) joint at the same level, and vice versa. Moreover, it also appeared that the influence of the magnitude of preloads on the facet force was less important than that due to the various postures. In addition, the inherent geometric asymmetry that exists in the model or coupled motion in the spine is a possible influencing factor with regard to the results, and this should be considered carefully in future studies.

## Competing interests

The authors declare that they have no competing interests.

## Authors' contributions

CSK participated in the study design, in collecting the data and drafting of the manuscript. HTH, RML, and KYH participated in the study design. PCL and ZCZ participated in revising the manuscript. MLH advised and assisted drafting of the manuscript. All authors read and approved the final manuscript.

## Pre-publication history

The pre-publication history for this paper can be accessed here:

http://www.biomedcentral.com/1471-2474/11/151/prepub
